# Cardiac Arrest Secondary to Inferior ST-Segment-Elevation Myocardial Infarction in Patient with Paroxysmal Nocturnal Hemoglobinuria and COVID-19 Infection

**DOI:** 10.7759/cureus.36632

**Published:** 2023-03-24

**Authors:** Mohamed Salah Mohamed, Amir Mahmoud, Anas Hashem, Ali Abdelhay, Mallory Balmer-Swain

**Affiliations:** 1 Internal Medicine, Rochester Regional Health, Rochester, USA; 2 Cardiology, Rochester Regional Health, Rochester, USA

**Keywords:** complement five inhibitors, st-elevation myocardial infarction (stemi), in hospital cardiac arrest, covid 19, paroxysmal nocturnal hemoglobinuria (pnh)

## Abstract

Patients with paroxysmal nocturnal hemoglobinuria (PNH) have transient attacks of complement-mediated hemolysis and thrombosis that can be spontaneous or secondary to precipitating factors such as infections. We present a case of a 63-year-old male patient with a medical history of PNH who presented with typical chest pain, fever, cough, jaundice, and dark-colored urine. On examination, he was hemodynamically stable but had conjunctival icterus. A few minutes after presentation, the patient suffered a ventricular fibrillation cardiac arrest and then achieved a return of spontaneous circulation after receiving two defibrillator shocks. EKG showed inferior wall ST-segment elevation myocardial infarction. Labs showed hemoglobin of 6.4 g/dl, elevated cardiac markers, serum lactate dehydrogenase, and indirect bilirubin. Serum haptoglobin was < 1 mg/dl. His COVID-19 polymerase chain reaction test was positive. Immediately, the patient received 2 units of packed RBCs and underwent a coronary angiogram (CA), which revealed total proximal occlusion of the right coronary artery. He underwent successful percutaneous coronary intervention (PCI), and two drug-eluting stents were placed. His peripheral blood immunophenotyping and flow cytometry showed loss of glycosylphosphatidylinositol-linked antigens and decreased expression of CD 59/14/24. He was started on ravulizumab, a humanized monoclonal antibody complement five inhibitor. Both PNH and COVID-19 increase the risk of thrombosis. Endothelial injury and cytokine storm increase the risk of thrombosis in COVID-19 patients, whereas the activation of the coagulation system and the impairment of the fibrinolytic system by complement cascade leads to thrombosis in PNH patients. Regardless of which pathway leads to coronary artery thrombosis, CA and PCI can be life-saving.

## Introduction

Paroxysmal nocturnal hemoglobinuria (PNH) is a hematological disorder characterized by complement-mediated intravascular hemolysis and platelet activation, leading to an increased risk of thrombosis, venous more common than arterial [1,2]. Key features include hemolytic anemia and possible thrombocytopenia. Coronavirus is a large group of viruses, and severe acute respiratory syndrome coronavirus 2 (SARS-CoV-2) causes a predominantly respiratory illness called COVID-19 [3,4]. COVID-19 is also associated with an increased risk of thrombosis by multiple mechanisms including endothelial cell dysfunction, increased coagulation, and decreased fibrinolysis [5]. We present a rare case of cardiac arrest secondary to inferior ST-segment elevation myocardial infarction (STEMI) in a patient with PNH and COVID-19 Infection.

## Case presentation

**Figure 1:**
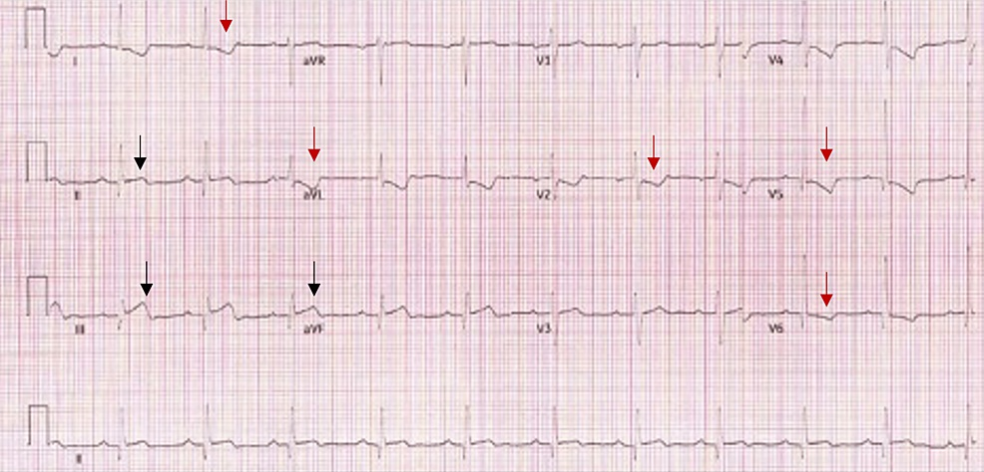
EKG showing inferior STEMI EKG showing ST-segment elevation in leads II, III, and aVF (black arrow) with ST-segment depression in leads I, aVL, V2, V5, and V6 (red arrow)

A 63-year-old male patient with a past medical history of PNH not on medical therapy presented to our emergency department with acute, crushing, retrosternal chest pain with radiation to the neck and left arm for one day. He also reported having fever, chills, fatigue, runny nose, jaundice, and dark-colored urine for a few days. In the emergency department, he was hemodynamically stable. Cardiac examination revealed normal S1 and S2 with no gallop, murmur, or rub. He then had a cardiac arrest secondary to ventricular fibrillation (VF). During the cardiopulmonary resuscitation, he received two defibrillator shocks after which he achieved a return of spontaneous circulation. His EKG showed ST-segment elevation in leads II, III, and aVF with ST-segment depression in leads I and aVL (Figure 1).

**Figure 2:**
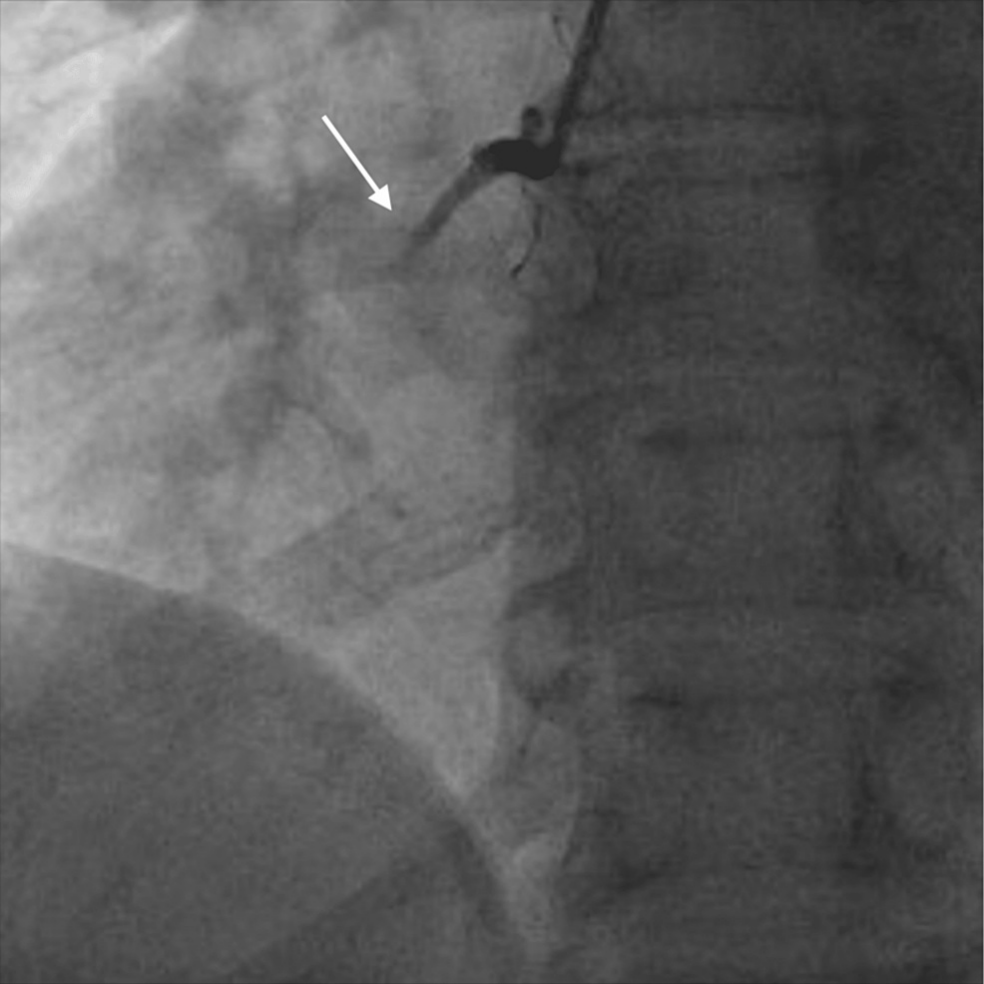
CA showing complete proximal occlusion of the RCA (white arrow)

His labs were significant for hemoglobin of 6.4 mg/dl, WBC count of 6.3 x 10^9^ L, platelet count of 176 x 10^9^ L, reticulocyte count of 19.6%, lactate dehydrogenase of 3912 U/L, haptoglobin of < 1 mg/dl, and high-sensitivity troponin of 1094 pg/mL. His COVID-19 polymerase chain reaction was positive. He received two units of packed RBC immediately and underwent an emergent coronary angiogram (CA), revealing complete proximal occlusion of the right coronary artery (RCA) (Figure 2).

**Figure 3:**
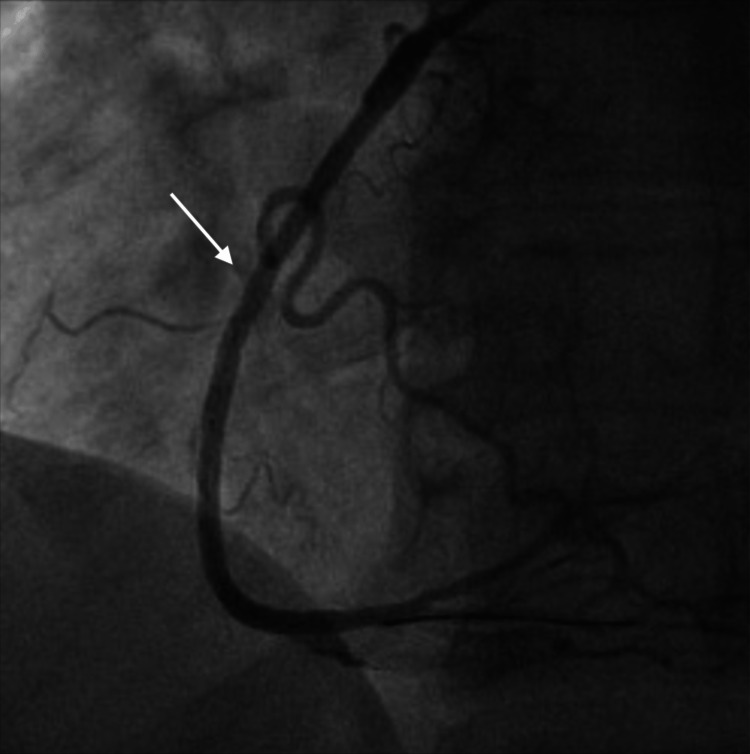
CA showing successful PCI of the RCA (white arrow)

He underwent successful percutaneous coronary intervention (PCI) with percutaneous transluminal coronary angioplasty and placement of two drug-eluting stents (Figure 3).

The patient was started on dual antiplatelet therapy with aspirin and clopidogrel post-PCI. He had no further arrhythmias for more than 48 hours. A transthoracic echocardiogram showed a left ventricle ejection fraction of 55% with normal left ventricle dimensions and a hypokinetic inferior wall. Further hematological workup revealed a negative direct Coombs test excluding autoimmune hemolytic anemia. Flow cytometry analysis revealed loss of glycosylphosphatidylinositol (GPI)-linked antigens, decreased expression of CD59 on 43% of CD235a-positive erythrocytes, reduced expression of CD14 on 98% of CD33-positive monocytes, and decreased expression of CD24 on 85% of CD15-positive granulocytes. These findings are indicative of PNH. He was started on ravulizumab, a humanized monoclonal antibody that inhibits complement five (C5), preventing further hemolysis and thrombosis. He was later seen in the cardiology clinic after one month, and he was completely symptom-free with no further episodes of chest pain or hemolysis.

## Discussion

PNH is a clonal hematopoietic stem cell disease that occurs due to a somatic mutation of the phosphatidylinositol glycan A (PIG-A) gene in bone marrow stem cells. Mutation of PIG-A results in the abnormal synthesis of GPI, causing a deficiency of all GPI-anchored proteins on the cell membrane of hematopoietic stem cells. CD55 and CD59 are two complement regulatory proteins that are deficient in PNH. The deficiency of these proteins results in intravascular hemolysis and platelet activation [2].

Due to complement-mediated platelet activation, patients with PNH have an increased risk of thromboembolism. In fact, thromboembolism is the most common cause of death in patients with PNH, approximately 40% to 65% [6]. PNH patients with thromboembolic complications have an increased risk of death by five- to fifteen-fold [6]. The mechanism of thromboembolism in PNH is complex. One of the mechanisms includes the activation of endothelial cells by the free hemoglobin released during complement-mediated intravascular hemolysis [1]. Another mechanism includes decreased GPI-anchored urokinase plasminogen activator on the platelets, which increases the risk of thrombosis. Thrombosis in PNH can occur at any site [6]. However, for unclear reasons, venous thrombosis is more common than arterial thrombosis [2]. The most common sites of venous thrombosis include hepatic vein thrombosis (causing Budd-Chiari syndrome), splenic vein thrombosis, and cerebral vein thrombosis (causing superior sagittal vein thrombosis). The most common sites of arterial thrombosis in patients with PNH include cerebral arteries (causing stroke) and coronary arteries (causing myocardial infarction) [6]. The gold-standard test for diagnosis of PNH is flow cytometry of RBC showing reduced expression of CD55 and CD59 [7]. Monoclonal antibody C5 inhibitors, such as eculizumab and ravulizumab, decrease the risk of intravascular hemolysis and thrombosis in patients with PNH by preventing the formation of membrane attack complex [1,8].

COVID-19 is independently associated with an increased risk of thromboembolism [9]. COVID-19 patients have a prothrombotic state due to endothelial cell dysfunction, excessive inflammation, increased coagulation, and decreased fibrinolysis. COVID-19 can result in both arterial and venous thromboembolism. However, venous thrombosis is more common than arterial thrombosis [10]. Developing thromboembolic manifestations in patients with COVID-19 disease is associated with increased mortality [10]. The increased thrombosis risk persists for up to 49 weeks after COVID-19 diagnosis [9]. Patients with STEMI and concomitant diagnosis of COVID-19 disease have a higher rate of in-hospital mortality compared to patients without COVID-19 disease [11]. According to the National Institute of Health COVID-19 treatment guidelines, the use of antiplatelet and anticoagulant therapies in COVID-19 patients to prevent arterial thrombosis is not recommended [12].

## Conclusions

Patients with PNH and COVID-19 infections have an increased risk of both venous and arterial thrombosis compared to the general population. Acute coronary artery thrombosis causing STEMI is one of the rare but most serious complications. Early and thorough cardiological evaluation for acute coronary syndrome in patients with PNH presenting with chest pain is essential and life-saving. Also, early initiation of monoclonal antibody C5 inhibitors, such as eculizumab and ravulizumab, can decrease the risk of intravascular hemolysis and thrombosis.
